# Description of two new species of the genus *Heterochelamon* Türkay & Dai, 1997 (Crustacea: Decapoda: Brachyura: Potamidae), from southern China

**DOI:** 10.7717/peerj.9565

**Published:** 2020-07-22

**Authors:** Song-Bo Wang, Yi-Yang Xu, Jie-Xin Zou

**Affiliations:** 1 Research Laboratory of Freshwater Crustacean Decapoda & Paragonimus, School of Basic Medical Sciences, Nanchang University, Nanchang City, Jiangxi Province, China; 2 Jiangxi Agricultural University, Nanchang City, Jiangxi Province, China; 3 Key Laboratory of Poyang Lake Environment and Resource Utilization, Ministry of Education, Nanchang University, Nanchang City, Jiangxi Province, China

**Keywords:** Freshwater crab, Taxonomy, *Heterochelamon*, Phylogenetic

## Abstract

This study describes two new species of freshwater crab of the genus *Heterochelamon* Türkay & Dai, 1997 from southern China, *H. huidongense* from Guangdong Province and *H. jinxiuense* from Guangxi Zhuang Autonomous Region. The two new species can be differentiated from congeners by characters derived from the shape of the epibranchial tooth, external orbital angle, cheliped proportions and structure of the male first gonopod. The present study brings the number of *Heterochelamon* species to seven. We used the mitochondrial 16S rRNA gene for a molecular analysis and the results are consistent with the morphological features that support the recognition of two new taxa.

## Introduction

Freshwater crabs are found in the tropics and subtropics in most parts of the world, occurring in aquatic habitats ranging from clear montane streams to lowland rivers and even in caves or tree holes (Dai, 1999; Ng, Guinot & Davie, 2008). Southern China, where mountains and rivers are abundant, provides a multitude of habitats for a large number of species and the large numbers of new taxa reported in recent years indicate that research on this group remains in a “discovery” phase (Yeo et al., 2008; Zhu, Naruse & Zhou, 2010; Naruse, Zhu & Zhou, 2013; Huang, Mao & Huang, 2014; Shih & Do, 2014; Huang, Shih & Mao, 2016; Huang, Shih & Ng, 2017; Huang, Ahyong & Shih, 2017; Ng, 2017; Huang, 2018; Huang, Shih & Ahyong, 2018; Huang, Wong & Ahyong, 2018; Wang, Huang & Zou, 2019; Wang, Zhou & Zou, 2019).

Currently, the freshwater crabs distributed in China are represented by 49 genera in the families Potamidae Ortmann, 1896 and Gecarcinucidae Alcock, 1910 (Chu et al., 2018; Chu, Wang & Sun, 2018; Huang, Shih & Ahyong, 2018; Huang, Huang & Shen, 2020). The potamid genus *Heterochelamon* was established by Türkay & Dai (1997) to accommodate *Potamon (Geothelphusa) purpureomanualis* Wu, 1934 (type species), theretofore assigned to *Malayapotamon*, and two new species, *H. guangxiense* and *H. yangshuoense*. Naruse, Zhu & Zhou (2013) further described two new species, *H. tessellatum* and *H. castanea*. The five known species exclusively occur in Guangxi Zhuang Autonomous Region (Türkay & Dai, 1997; Naruse, Zhu & Zhou, 2013).

In 2011, we collected specimens of a *Heterchelamon* species from Jinxiu, Guangxi, but the crabs were not identified at the time. It was not until 2018 that our research team identified the specimens as belonging to an undescribed species. In 2019, freshwater crab enthusiast Jia-Ming Tian collected some dark colored freshwater crabs from Huidong, Guangdong. We subsequently collected specimens of this species and found that they belong to another new species of *Heterochelamon*. We herein describe two new species of *Heterochelamon*, including the one from Guangdong Province, representing the first member of the genus from outside of Guangxi.

To study the phylogenetic relationships of species within *Heterochelamon*, we used the mitochondrial 16S rRNA gene data of four species from this genus, including the two new species reported in this paper. Molecular data supports the recognition of the two new species and their assignment to *Heterochelamon*.

## Material & Methods

Specimens were collected by Song-Bo Wang, Yi-Yang Xu, Jia-Ming Tian and Xi-Jiao Wei; preserved in 95% ethanol and deposited in the Department of Parasitology of the Medical College of Nanchang University, Jiangxi, China (NCU MCP), National Tropical Disease Research Center, Shanghai, China (TDRC). The abbreviations G1 and G2 are used for the male first gonopod and second gonopod, respectively. Carapace width and length are reported in millimeters. The terminology used primarily follows that of Dai (1999), Naruse, Zhu & Zhou (2013) and Davie, Guinot & Ng (2015).

Muscle tissue was extracted from the ambulatory legs and chelipeds, and total genomic DNA was extracted from the tissues using the Omega Tissue Kit following the manufacturer’s protocol. Mitochondrial 16S rRNA gene sequences were obtained by PCR amplification with the primers 1471 (5′-CCTGTTTANCAAAAACAT-3′) and 1472 (5′-AGATAGAAACCAACCTGG-3′) (Shih, Ng & Chang, 2004). The PCR procedure was as follows: 33 cycles of denaturation for 50 s at 94 °C, annealing for 40 s at 52 °C, and extension for 1 min at 72 °C, followed by a final extension for 10 min at 72 °C. Sequences were obtained by automated sequencing (ABI3730 automatic sequencer).

Sequences were aligned using MAFFT vers.7.355 (Nakamura et al., 2018) based on the GINS-I method and the selection of conserved regions with Gblocks 0.91b (Castresana, 2000). GTR+I+G was the best fit model for the sequence evolution of the 16S dataset, as determined with MrModeltest vers.2.2 (Nylander, 2005) and then selected based on the Akaike information criterion (AIC). MrBayes vers.3.2.6 (Ronquist et al., 2012) was used to construct a Bayesian inference (BI) tree. Four Markov chain Monte Carlo (MCMC) chains were run for 2,000,000 generations, with samples stored once every 1000 generations, discarding the first 25% as burnin. Tracer vers.1.6 (Rambaut et al., 2014) was used to check effective sample size (ESS) values (all of greater than 200). Simultaneously with the BI analysis, we used MEGA vers.X.0 (Kumar et al., 2018) to select the best evolutionary model for maximum likelihood (ML) analysis, which was the HKY+I+G model based on the Bayesian information criterion (BIC). The ML tree was built after 1000 bootstrap replicates by using MEGA vers.X.0 (Kumar et al., 2018).

The electronic version of this article in portable document format will represent a published work according to the International Commission on Zoological Nomenclature (ICZN), and hence the new names contained in the electronic version are effectively published under that Code from the electronic edition alone. This published work and the nomenclatural acts it contains have been registered in ZooBank, the online registration system for the ICZN. The ZooBank LSIDs (Life Science Identifiers) can be resolved and the associated information viewed through any standard web browser by appending the LSID to the prefix http://zoobank.org/. The LSID for this publication is: urn:lsid:zoobank.org:pub: F680C21E-0BED-4724-BA62-1174336F433F. The online version of this work is archived and available from the following digital repositories: Peer J, PubMed Central, and CLOCKSS.

## Results

### Systematics

**Table:** 

**Figure 1:**
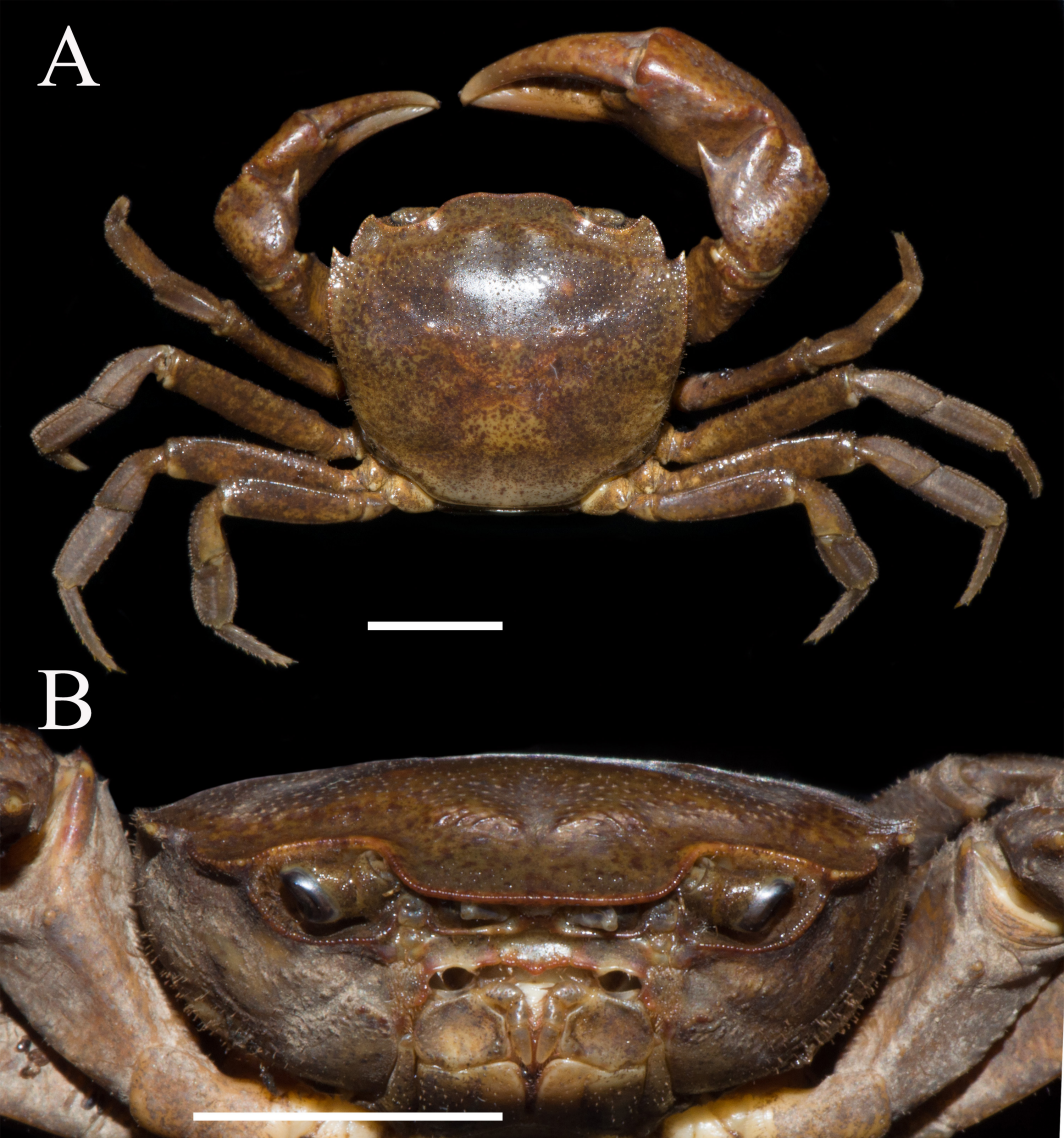
*Heterochelamon huidongense.* n. sp. Holotype male (25.8 × 23.1 mm) (NCU MCP 423601). (A) Overall habitus; (B) frontal view of the cephalothorax. Scales = one cm. Photo credit: Song-Bo Wang.

**Figure 2:**
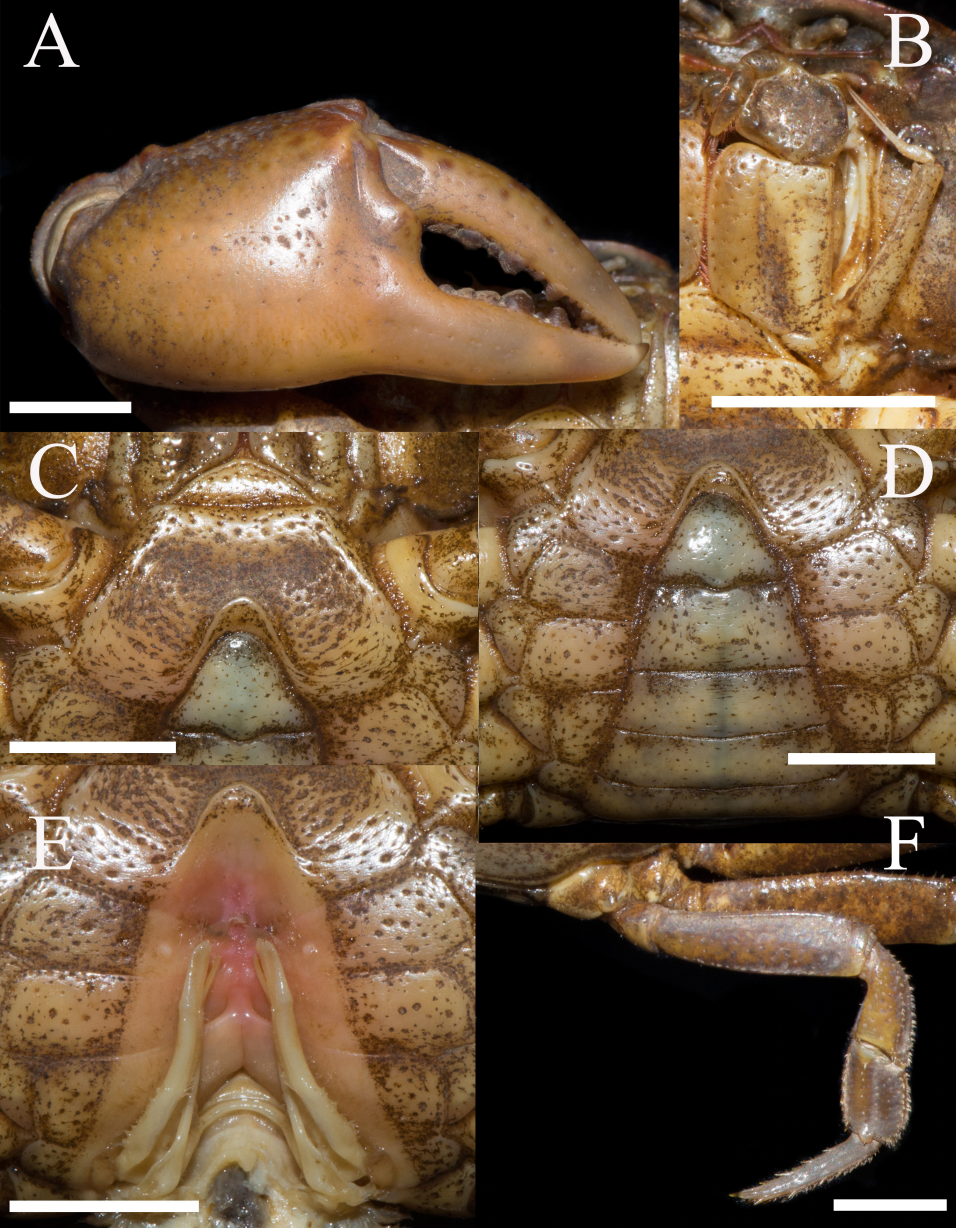
*Heterochelamon huidongense* n. sp. Holotype male (25.8 × 23.1 mm) (NCU MCP 423601). (A) Outer view of larger cheliped; (B) left third maxilliped; (C) ventral view of anterior thoracic sternum; (D) ventral view of pleon; (E) ventral view of sterno-pleonal cavity with right G1 in situ; (F) right fourth ambulatory leg. Scales = 0.5 cm. Photo credit: Song-Bo Wang.

**Figure 3:**
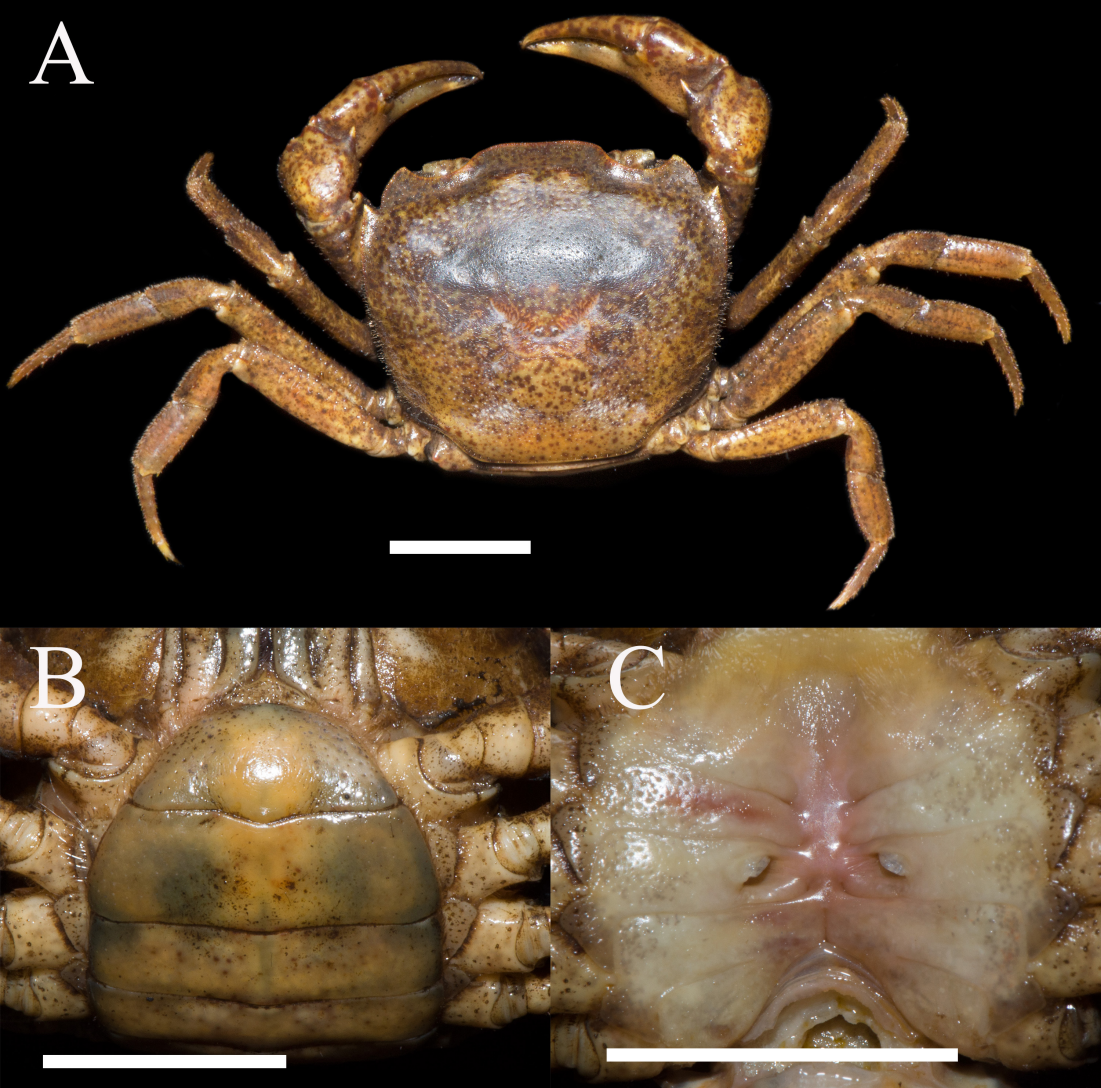
*Heterochelamon huidongense.* n. sp. Paratype female (26.1 × 23.5 mm) (NCU MCP 423603). (A) Overall habitus; (B) ventral view of pleon; (C) vulvae. Scales = one cm. Photo credit: Song-Bo Wang.

**Figure 4:**
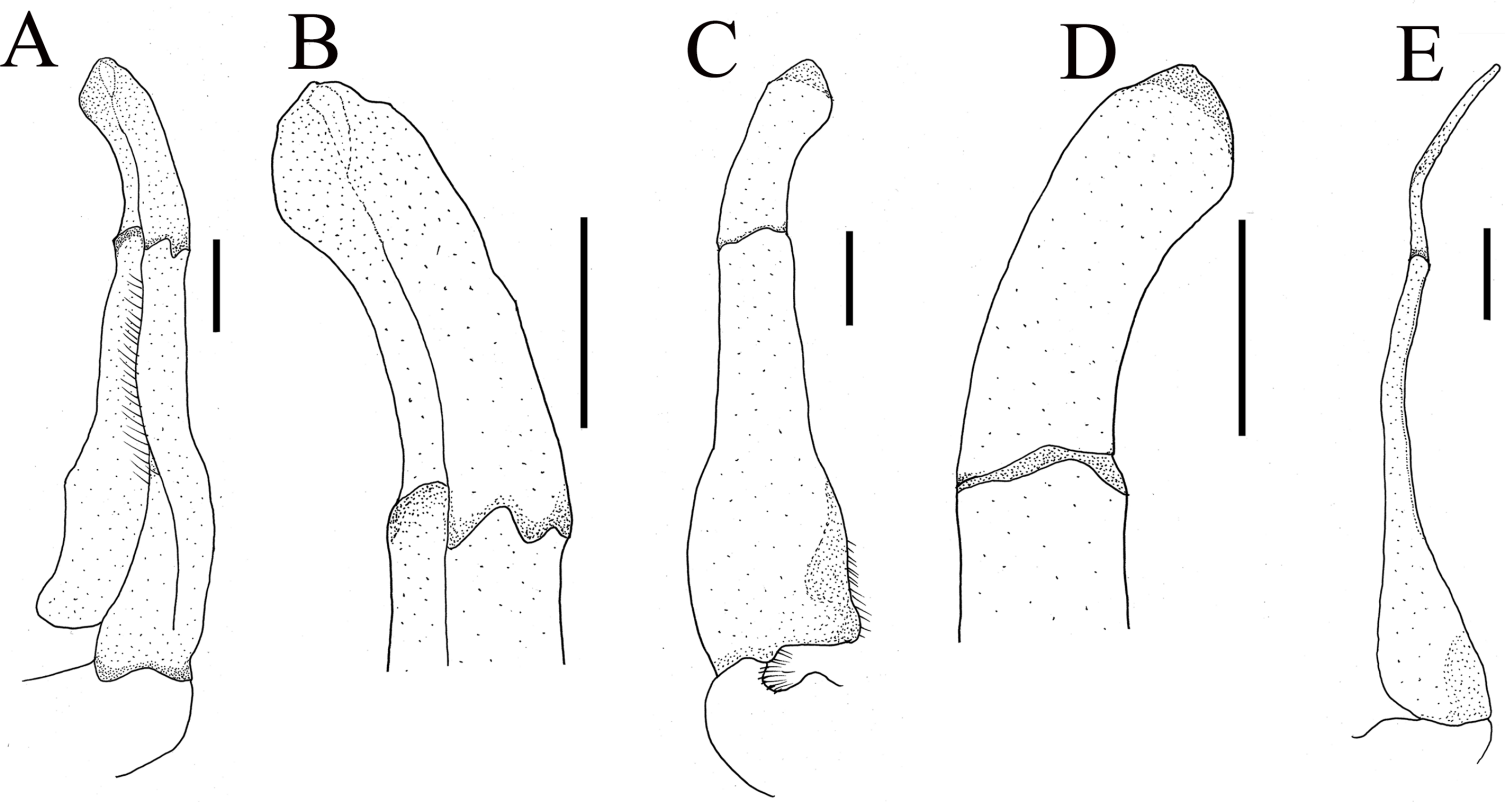
Gonopods of holotype of *Heterochelamon huidongense* n. sp. (A) Ventral view of the left G1; (B) ventral view of the terminal segment of left G1; (C) dorsal view of the left G1; (D) dorsal view of the terminal segment of left G1; (E) ventral view of the left G2. Scales = one mm. Photo credit: Song-Bo Wang.

**Family Potamidae Ortmann, 1896**
***Heterochelamon*Türkay & Dai, 1997**
***Heterochelamon huidongense*n. sp. (Figs. 1–4)**
urn:lsid:zoobank.org: act: 0F67FA91-6734-4800-9C03-76668635A1E2

**Material examined.** Holotype: male (25.8 × 23.1 mm) (NCU MCP 423601), Xinaobei Village (22°55′14.59″N 114°33′51.85″E, 47 m asl.), Baihua Town, Huidong County, Huizhou City, Guangdong Province, coll. Song-Bo Wang, Yi-Yang Xu and Jia-Ming Tian, 6th Jul. 2019. Paratypes: 3 males (30.0 × 25.8 mm, 26.2 × 23.1 mm, 21.3 × 18.6 mm) (NCU MCP 423602, TDRC 002005, TDRC 002006) and 2 females (26.1 × 23.5 mm, 20.6 × 18.5 mm) (NCU MCP 423603, TDRC 002007), same data as holotype. Others: 5 males (29.6 × 25.4 mm, 26.4 × 23.5 mm, 25.1 × 22.3 mm, 28.4 × 24.5 mm, 21.3 × 18.9 mm; NCU MCP 423606, NCU MCP 423607, NCU MCP 423608, NCU MCP 423609, NCU MCP 423610) and 2 females (21.1 × 19.1 mm, 14.5 × 12.5 mm; NCU MCP 423612, NCU MCP 423613), same data as holotype.

**Comparative material.** *Heterochelamon yangshuoense* Türkay & Dai, 1997: Holotype, male, IZCAS CB 05102, Yangshuo County, Guilin City, Guangxi Zhuang Autonomous Region, 8th May 1975. *H. guangxiense* Türkay & Dai, 1993: Holotype, male, IZCAS CB 01336, Guangxi Zhuang Autonomous Region, May 1974. *H. purpureomanualis* Türkay & Dai, 1997: not holotype, male, IZCAS CB, Yao Mountain, Xiuren Town, Lipu County, Guilin City, Guangxi Zhuang Autonomous Region, 1938. *H. tessellatum* Naruse, Zhu & Zhou, 2013: Holotype, male, NCU MCP 2012.0003, Dong Men Zhuang, Shuangluo Village, Sanli Town, Shanglin County, Guangxi Zhuang Autonomous Region, 14th Aug. 2006, Paratypes, 1 male, NCU MCP 2012.0004, 1 female, NCHUZOOL 13572, same data as holotype. *H. castanea* Naruse, Zhu & Zhou, 2013: Holotype: male, NCU MCP 2012.0001, Lituan Village, Baiming Town, Liujiang County, Liuzhou City, Guangxi Zhuang Autonomous Region, Aug. 2006, Paratype: 1 female, NCU MCP 2012. 0002, same data as holotype.

**Diagnosis.** Carapace subquadrate, dorsal surface covered with scattered minute pits, regions not clearly demarcated. External orbital angle triangular, sharp; postorbital cristae inconspicuous. Epibranchial tooth very sharp, distinct. Anterolateral margin cristate. Suborbital, pterygostomial regions smooth. Median lobe of epistome posterior margin protruded slightly. Third maxilliped exopod exceeding posterior margin of merus, with long slender flagellum. Chelipeds unequal, cutting edges of finger of larger cheliped with blunt teeth, with narrow gap when fingers closed. Ambulatory legs slender. Male telson triangular, lateral margins slightly concave. Female vulva located at middle of sternite 6. G1 terminal segment flat and medially bent, not tapered distally, apex rounded, reaching pleonal locking tubercle on mid length of sternite 5 but not exceeding sternites 4/5 suture.

**Description.** Carapace subquadrate, width about 1.1 times as length (*n* = 13), regions not clearly demarcated; dorsal surface convex longitudinally, smooth (Figs. 1A, 3A). External orbital angle triangular, sharp, separated from anterolateral margin by U-shaped notch; postorbital cristae inconspicuous, postfrontal lobe slightly convex, separated from each other; cervical groove indistinct; H-shaped gastric groove indiscernible (Figs. 1A and 3A). Epibranchial tooth very sharp, distinct; anterolateral margin cristate, straight, lined with 7–8 granules, shorter than posterolateral margin; posterolateral surface smooth (Figs. 1A, 3A). Frontal, supra-, infraorbital margins cristate, lined with indistinct granules; suborbital, pterygostomial regions smooth, with a few round granules (Fig. 1B). Epistome posterior margin distinctly cristate, median lobe low, triangular, lateral margins slightly sinuous (Fig. 1B).

Third maxilliped exopod exceeding posterior margin of merus, with long slender flagellum; merus about 1.2 times as broad as long, slightly depressed medially; ischium about 1.4 times as long as broad, with distinct median sulcus (Fig. 2B).

Male chelipeds unequal (Fig. 1A). Merus surface smooth without any pits; carpus with inner angle produced into strong spine, surface sparsely covered with pits (Fig. 1A). Cutting edges of larger cheliped fingers with row of large blunt teeth, with narrow gap when fingers closed (Fig. 2A). Ambulatory legs slender; third legs longest when stretched laterally, fourth ambulatory leg propodus about 1.6 times as long as broad, slightly shorter than dactylus, with scattered thorn-like setae (Fig. 2E).

Male thoracic sternites 2/3 demarcated by distinct suture, sternites 3/4 demarcated by shallow groove (Fig. 2C). Male sterno-pleonal cavity deep, reaching anteriorly to mid-length of thoracic sternite 4; median groove between sternites 7/8 long (Fig. 2E). Male pleon triangular, somites 3–6 gradually decreasing in width, increasing in length in males; somite 3 widest; somite 6 trapezoidal, margins slightly oblique (Fig. 2D). Telson triangular with rounded apex, lateral margin slightly concave (Fig. 2C). Female pleon broadly ovate (Fig. 3B); vulva small, ovate, located at middle of sternite 6, opening inwards, posterior margin slightly bulged (Fig. 3C).

G1 slender; terminal segment flat, bent medially, not tapered distally, distal part rounded; exceeding pleonal locking tubercle on mid length of sternite 5, not reaching suture between sternites 4/5 (Figs. 4A–4D, 2F). Distinct boundary between terminal segment and subterminal segment, latter length about 2.5 times as former, groove for G2 in ventral surface; G2 basal segment triangular, length about 2.1 times distal segment (Fig. 4).

**Etymology.** The species is named after the type locality, Huidong County, Huizhou City, Guangdong Province.

**Distribution.** The new species is known only from the type locality presently, Huidong County, Huizhou City, Guangdong Province, southern China.

**Figure 5:**
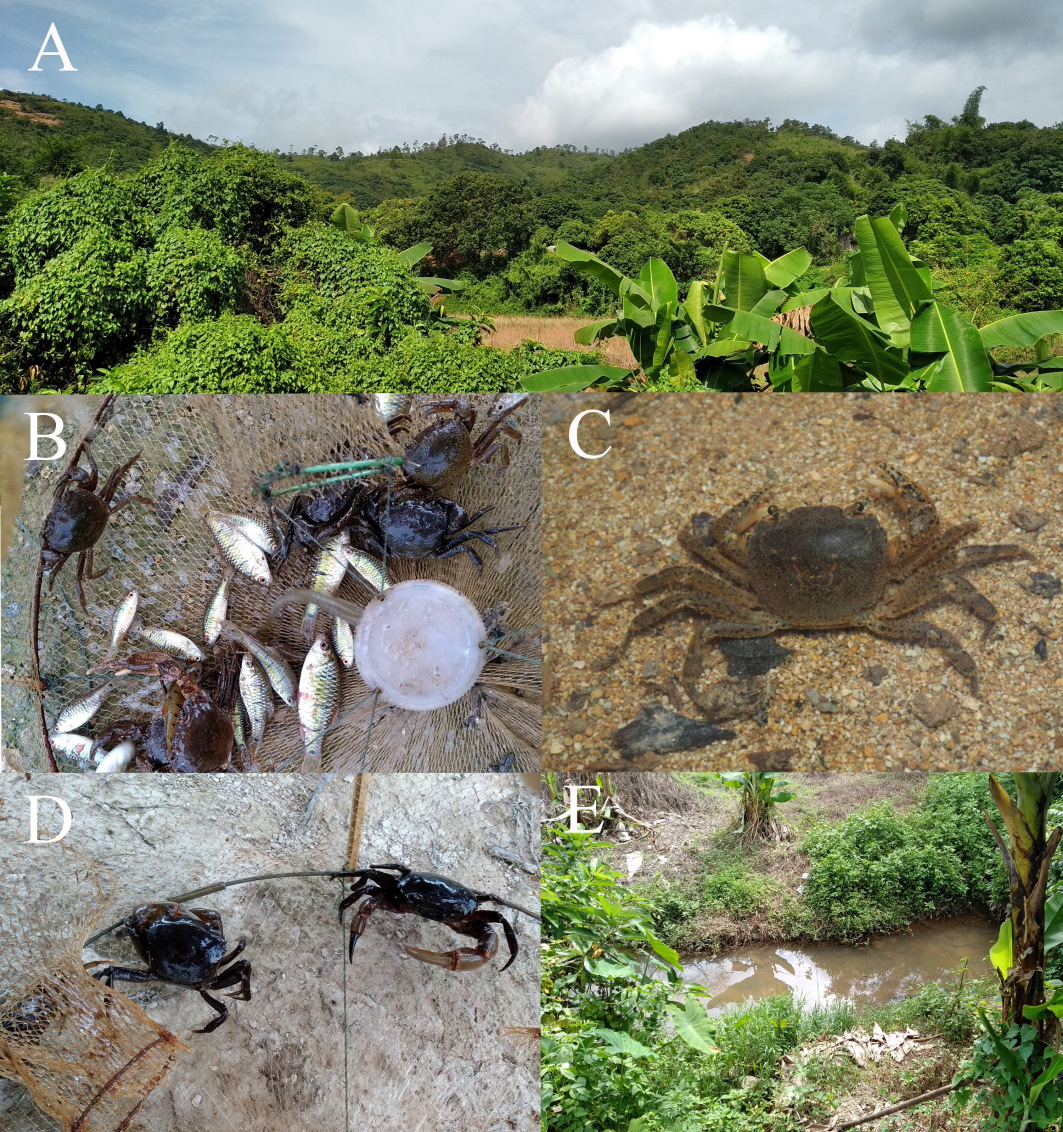
Habitat Environment of *Heterochelamon huidongense* n. sp. (A) Surrounding environment of the collection point; (B) living specimen of *H. huidongense* n. sp. collected with a fish cage; (C) *H. huidongense* n. sp. in the water; (D) living specimen of *Somanniathelphusa* Bott, 1968, collected with a fish cage; (E) collection point. Photo credit: A, B, D, E from Song-Bo Wang and C from Chao Huang.

**Ecology.** Specimens of the new species were collected at three sites along a river using fish cages or by hand (Figs. 5B, 5C). The water of the river is relatively clear, and the water flow is slow. The width of the river is approximately 1–3 m and the depth is approximately 0.2–0.3 m. The river bed is mainly sand and soil (Fig. 5E). There are many shrubs and low hills around the river, many households located dozens of meters away from the river and some vegetable gardens along the river (Fig. 5A). At one of the collection points, we also collected specimens of the gecarcinucid *Somanniathelphusa sinensis* H. Milne-Edwards, 1853 (Fig. 5D), which we believe coexists with the new species in the river.

**Remarks.** Compared to congeners, *Heterochelamon huidongense* n. sp. can easily be separated by its G1 not reaching sternites 4/5 suture in situ and terminal segment being medially bent (Figs. 2E and 4A, Table 1) (versus reaching sternites 4/5 suture and straight in congeners, except *H. tessellatum* which has G1 that also does not reach the sternites 4/5 suture but is strongly bent proximally), as well as a combination of characters: very sharp epibranchial tooth , triangular external orbital angle, slightly convex anterolateral margins (Fig. 1A), triangular male telson (Fig. 2C), and inward opening female vulva (Fig. 3C). For detailed differences between this new species and congeners, see Table 1.

**Table 1:** Differences between the species of Heterochelamon Türkay & Dai, 1997.

	*H. huidongense*	*H. jinxiuense*	*H. tessellatum*	*H. castanea*	*H. purpureomanualis*^a^	*H. purpureomanualis*^b^	*H. guangxiense*	*H. yangshuoense*
External orbital angle	Triangular (Fig. 1A)	Blunt (Fig. 6A)	Acutely triangular (cf. Naruse, Zhu & Zhou, 2013: Fig. 1A)	Triangular (cf. Naruse, Zhu & Zhou, 2013: Fig. 4A)	Acutely triangular (cf. Wu, 1934: Fig. 2)	Triangular (cf. Türkay & Dai, 1997)	Blunt (cf. Türkay & Dai, 1997)	Triangular (cf. Türkay & Dai, 1997)
Epibranchial tooth	Very sharp, separated from external orbital angle by broad notch (Fig. 1A)	Blunt, separated from external orbital angle by narrow notch (Fig. 6A)	Very sharp, separated from external orbital angle by broad notch (cf. Naruse, Zhu & Zhou, 2013: Fig. 1A)	Very sharp, separated from external orbital angle by narrow notch (cf. Naruse, Zhu & Zhou, 2013: Fig. 4A)	Very sharp, separated from external orbital angle by narrow notch (cf. Wu, 1934: Fig. 2)	Blunt, separated from external orbital angle by narrow notch (cf. Türkay & Dai, 1997)	Very sharp, separated from external orbital angle by broad notch (cf. Türkay & Dai, 1997)	Very sharp, separated from external orbital angle by broad notch (cf. Türkay & Dai, 1997)
Adult male chelipeds	Unequal (Fig. 1A)	Strongly unequal (Fig. 6A)	Strongly unequal (cf. Naruse, Zhu & Zhou, 2013: Fig. 1A)	Unequal (cf. Naruse, Zhu & Zhou, 2013: Fig. 4A)	Strongly unequal (cf. Wu, 1934: Fig. 2)	Strongly unequal (cf. Türkay & Dai, 1997)	Unequal (cf. Türkay & Dai, 1997)	Strongly unequal (cf. Türkay & Dai, 1997)
Gap of adult male major chela fingers when closed	Narrow, wedge-shaped (Fig. 2A)	Medium, teardrop-shaped (Fig. 7A)	Very broad, oblong (cf. Naruse, Zhu & Zhou, 2013: Fig. 2B)	Narrow, wedge-shaped (cf. (Naruse, Zhu & Zhou, 2013): Fig. 5B)	Very broad, oblong (cf. Wu, 1934: Fig. 2)	Very broad, oblong (cf. Türkay & Dai, 1997)	Narrow, wedge-shaped (cf. Türkay & Dai, 1997)	Very broad, oblong (cf. Türkay & Dai, 1997: fig. 6-2)
G1 in situ	Not reaching sternites 4/5 suture (Fig. 2E)	Reaching sternites 4/5 suture (Fig. 8E)	Not reaching sternites 4/5 suture (cf. Naruse, Zhu & Zhou, 2013: Fig. 2A)	Reaching sternites 4/5 suture (cf. Naruse, Zhu & Zhou, 2013)	No information	Reaching sternites 4/5 suture (cf. Türkay & Dai, 1997: fig. 4-3)	Reaching sternites 4/5 suture (cf. Türkay & Dai, 1997: fig. 5-3)	Reaching sternites 4/5 suture (cf. Türkay & Dai, 1997: fig. 6-4)
Terminal segment of G1	Flat, bent in middle, not tapered distally, distal part rounded (Fig. 4)	Rod-like, straight, tapered distally, distal part sharp, bent slightly at distal (Fig. 9)	Knuckle-shaped, strongly bent proximally, widening distally, widest part more than twice as wide as base (cf. Naruse, Zhu & Zhou, 2013: Fig. 3A)	Rod-like, straight, not tapered distally, distal part rounded and bent slightly (cf. Naruse, Zhu & Zhou, 2013: Fig. 6A)	No information	Rod-like, straight, not tapered distally, distal part protruding slightly in inner edge (cf. Türkay & Dai, 1997: fig. 4-4)	Rod-like, slightly bent, not tapered distally, distal part protruding in inner edge (cf. Türkay & Dai, 1997: fig. 5-4)	Knuckle-shaped, straight, widening distally, widest part more than twice as wide as base (cf. Türkay & Dai, 1997: fig. 6-5)

**Notes.**

a Recorded by Wu (1934) from Luocheng County, Guangxi Zhuang Autonomous Region, China.

b Recorded by Türkay & Dai (1997) cf. (Wu, 1934) from Xiuren County, Guangxi Zhuang Autonomous Region, China.

**Table:** 

**Figure 6:**
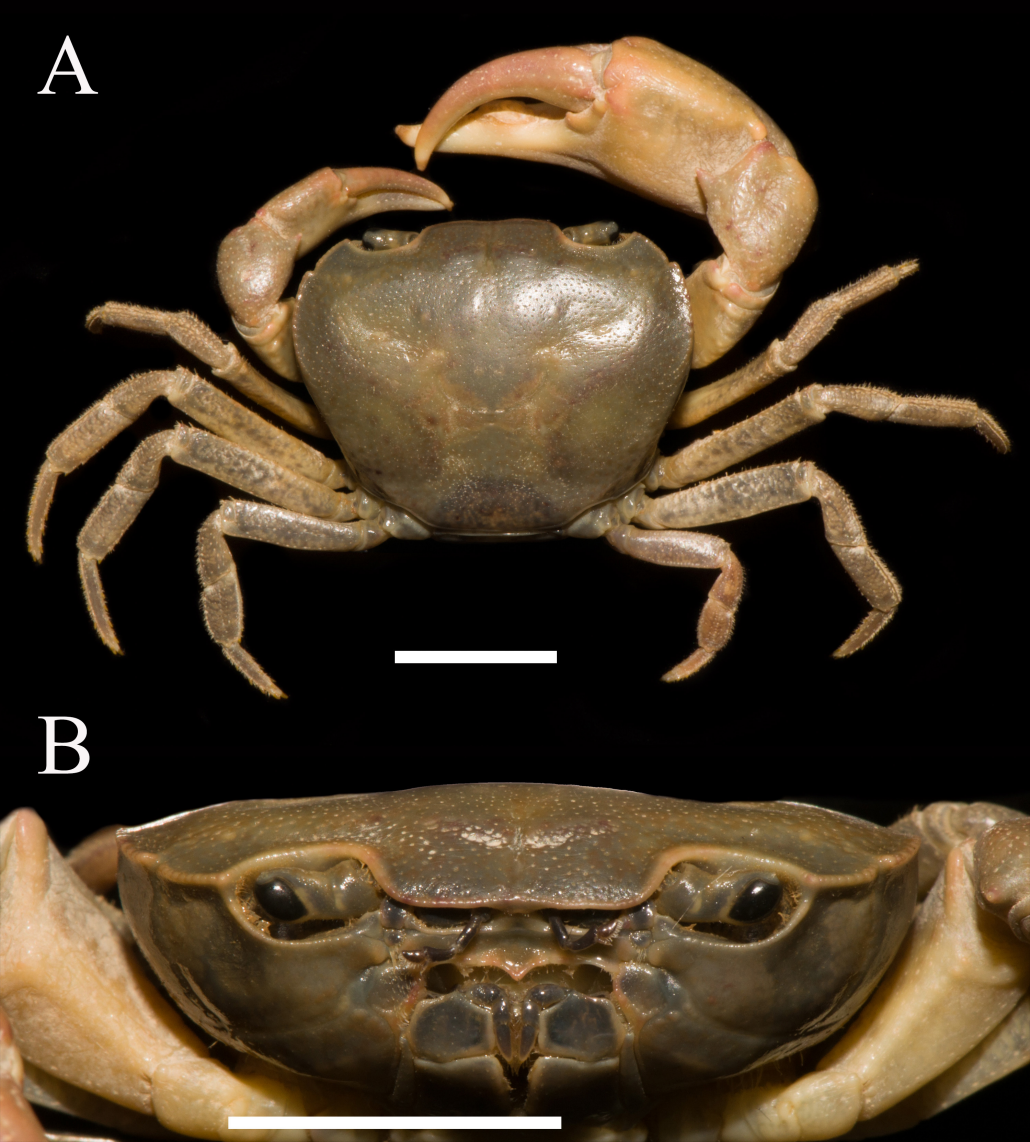
*Heterochelamon jinxiuense.* n. sp. Holotype male (24.6 × 19.8 mm) (NCU MCP 342001). (A) Overall habitus; (B) frontal view of the cephalothorax. Scales = one cm. Photo credit: Song-Bo Wang.

**Figure 7:**
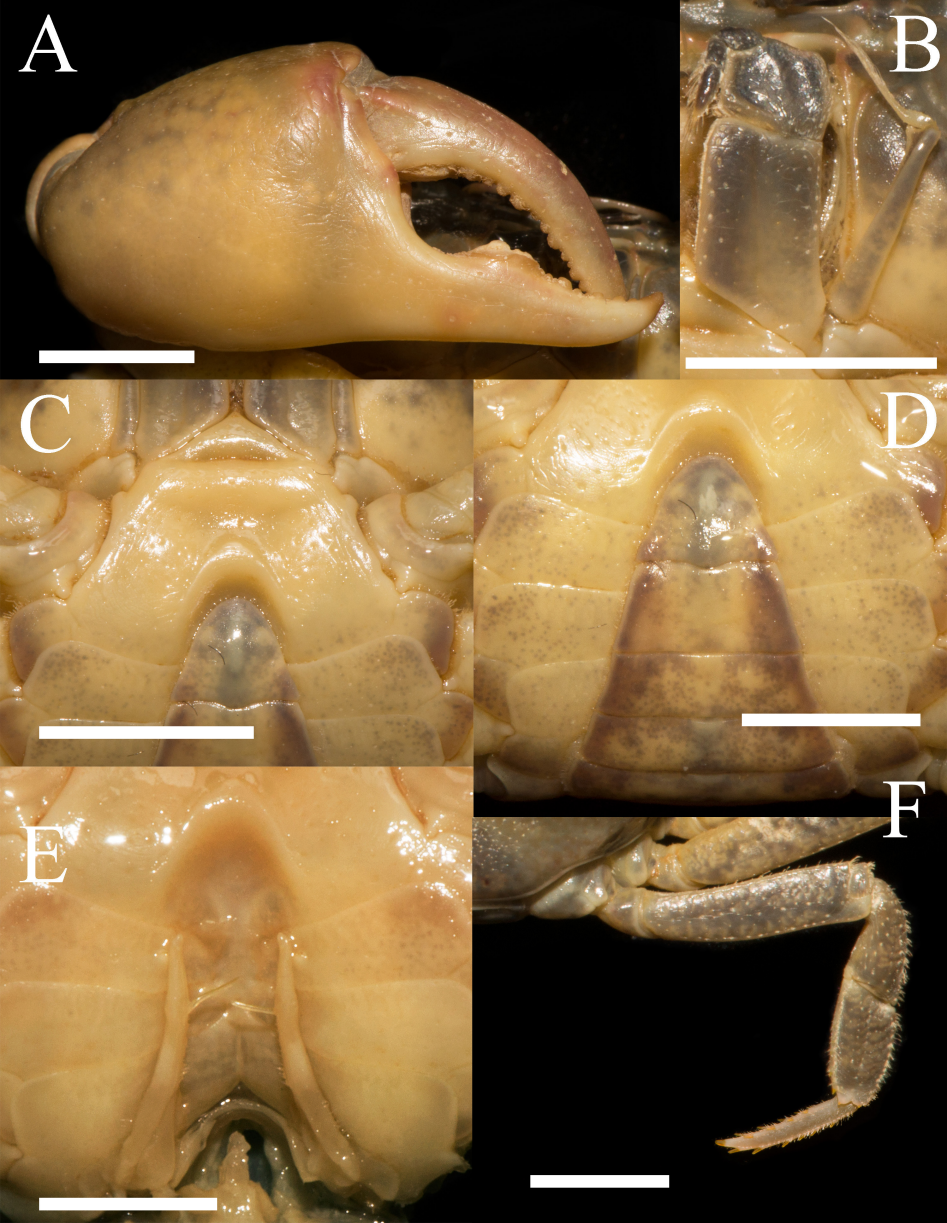
*Heterochelamon jinxiuense.* n. sp. Holotype male (24.6 × 19.8 mm) (NCU MCP 342001). (A) Outer view of larger cheliped; (B) left third maxilliped; (C) ventral view of anterior thoracic sternum; (D) ventral view of pleon; (E) ventral view of sterno-pleonal cavity with right G1 in situ; (F) right fourth ambulatory leg. Scales = 0.5 cm. Photo credit: Song-Bo Wang.

**Figure 8:**
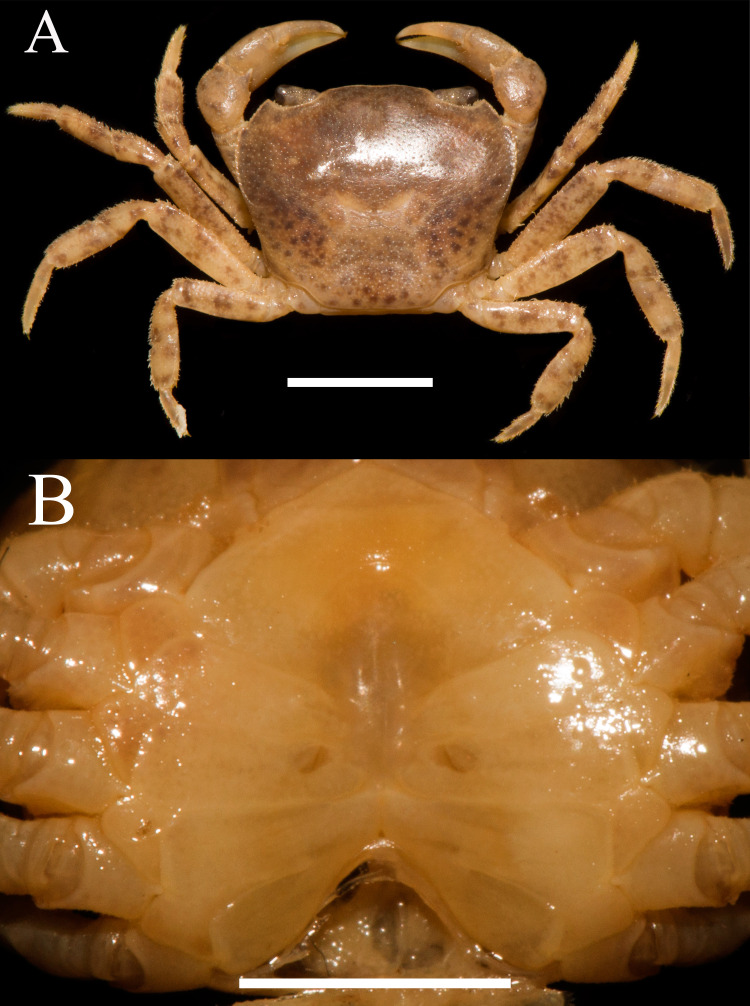
*Heterochelamon jinxiuense.* n. sp. Paratype female (12.9 × 10.4 mm) (NCU MCP 342003). (A) Overall habitus; (B) ventral view of vulvae. Scales = one cm. Photo credit: Song-Bo Wang.

***Heterochelamon jinxiuense*n. sp. (Figs. 5–8)**
urn:lsid:zoobank.org:act:5D34B999-78C7-4522-9F74-E872A34B7462

**Material examined.** Holotype: 1 male (24.6 × 19.8 mm) (NCU MCP 342001), from Tongfu Village (24°17′10.89″N115°5′17.28″E, 267 m asl.), Toupai Town, Jinxiu Yao Autonomous County, Laibin City, Guangxi Zhuang Autonomous Region, coll. Xi-Jiao Wei, 8th Jul. 2011. Paratypes: 3 males (20.3 × 15.8 mm, 15.8 × 12.6 mm, 17.8 × 14.8 mm) (NCU MCP 342002, TDRC 002008, TDRC 002009) and 1 female (12.9 × 10.4 mm) (NCU MCP 342003), same data as holotype. Other: 1 male (14.1 × 11.4 mm) (NCU MCP 342006), same data as holotype.

**Comparative material.** See for *Heterochelamon huidongense* n. sp.

**Diagnosis.** Carapace trapezoidal, flat, surface covered with scattered minute pits. External orbital angle blunt, postorbital cristae, postfrontal lobe indiscernible. Epibranchial tooth blunt, distinct; anterolateral margin cristate, slightly convex. Suborbital, pterygostomial regions very smooth. Median lobe of epistome posterior margin distinctly protruding. Third maxilliped exopod with long flagellum. Chelipeds strongly unequal in adult males, surface smooth, lower cutting edge of major cheliped finger with very large, blunt teeth, with medium gap when fingers closed. Ambulatory legs slender. Thoracic sternites 2/3 demarcated by distinct deep suture, anterior part of sternite 3 convex. Male sterno-pleonal cavity deep, narrow. Male telson triangular, lateral margin straight. Female vulva reaching sternites 5/6 suture. Male G1 terminal segment rod-like, tapered distally, subdistal segment suddenly inwardly bent, reaching sternites 4/5 suture.

**Figure 9:**
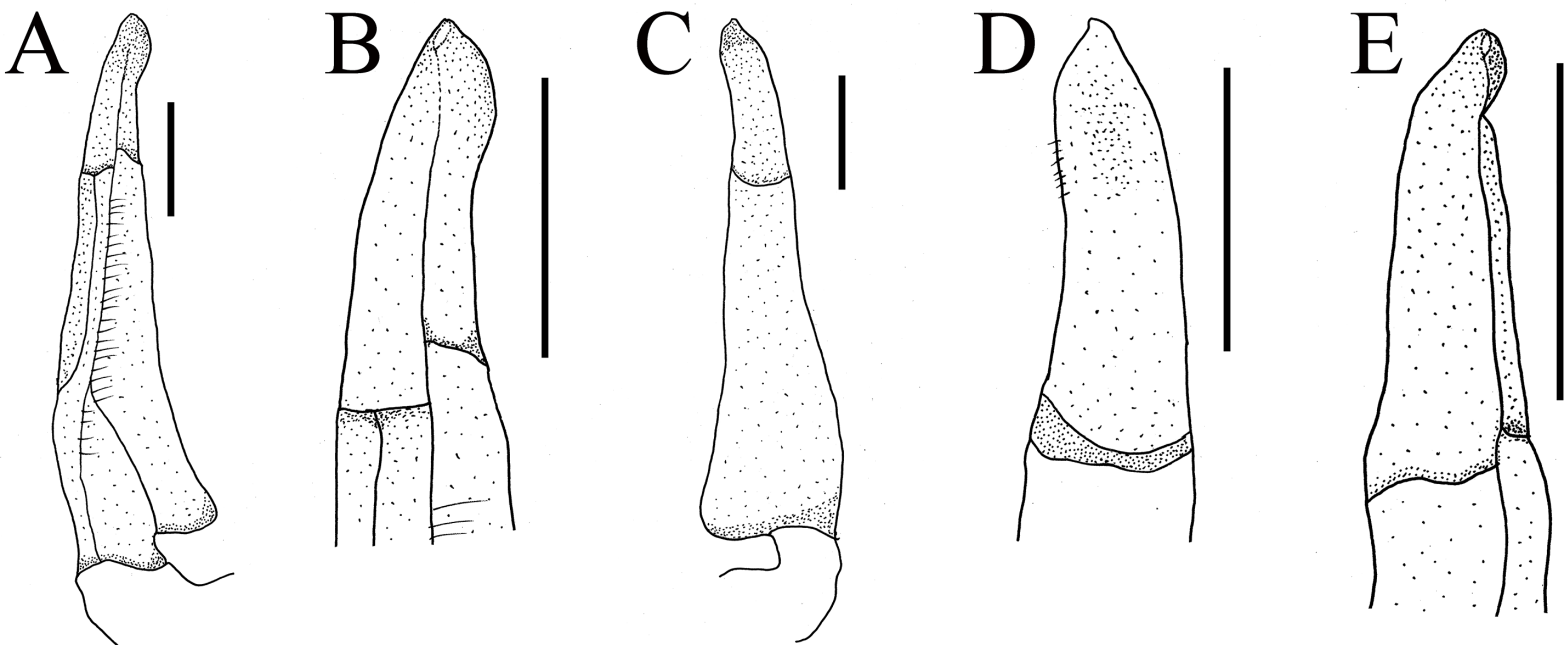
The first gonopods of holotype of *Heterochelamon jinxiuense* n. sp. (A) Ventral view of the right G1; (B) ventral view of the terminal segment of right G1; (C) dorsal view of the right G1; (D) dorsal view of the terminal segment of right G1; (E) side view of the right G1. Scales = one mm. Photo credit: Song-Bo Wang.

**Description.** Carapace trapezoidal, flat, width about 1.3 times as length (*n* = 6), regions not clearly demarcated; dorsal surface slightly convex, covered with inconspicuous pits (Figs. 6A, 8A). External orbital angle blunt, separated from anterolateral margin by shallow notch; postorbital cristae inconspicuous, postfrontal lobe indistinct; cervical groove indistinct; H-shaped gastric groove indiscernible (Figs. 6A, 8A). Epibranchial tooth blunt, distinct; anterolateral margin cristate, slightly convex, lined with several granules, shorter than posterolateral margin; posterolateral surface smooth (Figs. 6A, 8A). Frontal and orbital margins cristate, lined with indistinct granules, supra-, infraorbital margins glazed; suborbital, pterygostomial regions very smooth, not covered with granules (Fig. 6B). Epistome posterior margin distinctly cristate, median lobe triangular, distinctly protruding, lateral margins almost straight (Fig. 6B).

Third maxilliped exopod exceeding posterior margin of merus, with slender flagellum; merus about 1.2 times as broad as long, with median depression; ischium about 1.4 times as long as broad, longitudinal median sulcus distinct (Fig. 7B).

Chelipeds strongly unequal in adult males (Fig. 7A). Merus surface smooth without any pits; carpus inner angle forming stout spine, surface not covered with pits (Fig. 1A). Cutting edge of larger cheliped fixed finger with large rounded teeth, movable finger cutting edge lined with several small teeth, with medium gap when fingers closed (Fig. 7A). Ambulatory legs slender; third legs longest, the fourth ambulatory leg propodus about 2.0 times as long as broad, equal to dactylus in length (Figs. 6A, 7F).

Male thoracic sternites 2/3 demarcated by distinct deep suture, anterior part of sternite 3 convex, sternites 3/4 suture discernible as shallow groove, sterno-pleonal cavity reaching imaginary line joining posterior third of cheliped coxae (Fig. 7C). Male sterno-pleonal cavity deep, narrow; median longitudinal groove between sternites 7/8 medium-length (Fig. 7E). Male pleon triangular, third somite widest; somite 6 trapezoidal; somites 3–6 gradually decreasing in width (Fig. 7D). Telson triangular with rounded apex, lateral margin slightly convex (Fig. 7D). Female vulva very small, ovate, reaching sternites 5/6 suture, located at upper of sternite 6 (Fig. 8B).

Male G1 slender; terminal segment rod-like, almost straight, tapered distally; apex sharp, abruptly bent inwards subdistally; reaching beyond pleonal locking tubercle on mid length of sternite 5, to suture between sternites 4/5 (Figs. 7E, 9). Boundary between terminal segment and subterminal segment clear, latter length about 2.4 times as former, groove for G2 in ventral surface, G2 destroyed during dissection (Fig. 9).

**Etymology.** The species is named after the type locality, Jinxiu Yao Autonomous County, Laibin City, Guangxi Zhuang Autonomous Region.

**Distribution.** The new species is known only from the type locality presently, Jinxiu Yao Autonomous County, Laibin City, Guangxi Zhuang Autonomous Region, southern China.

**Ecology.** The collection site is a small river running behind Tongfu village. The river is mostly surrounded by dwarf mountains and many kinds of fruit trees. The river is approximately 2 m in width, the water depth is approximately 0.4 m, the riverbed mainly consists of sand and gravel and the water quality is good.

### Remarks

*Heterochelamon jinxiuense* n. sp. can easily be differentiated from its congeners by its distally tapered G1 (Fig. 9, Table 1) (versus widening distally or not tapered distally in congeners), blunt external orbital angle (Fig. 6A, Table 1) (versus triangular or acutely triangular in congeners, except that of *H. guangxiense* which is also blunt), strongly unequal chelipeds in adult males and medium gap when male major chela fingers closed (Fig. 7A, Table 1) (versus strongly unequal in *H. tessellatum*, *H. purpureomanualis* and *H. yangshuoense*, but fingers with very broad and oblong gap when closed; other species unequal with narrow gap when closed). The detailed differences between this new species and congeners are also presented in Table 1.

The new species was collected in 2011, of which the material consisted of five males (one adult and four immature) and one female. The G2 of the only mature male had been destroyed by the time we examined the specimens. We were also unable to find this species in a subsequent collection trip. As of such, the G2 is not described here.

### Phylogenetic analyses

**Figure 10:**
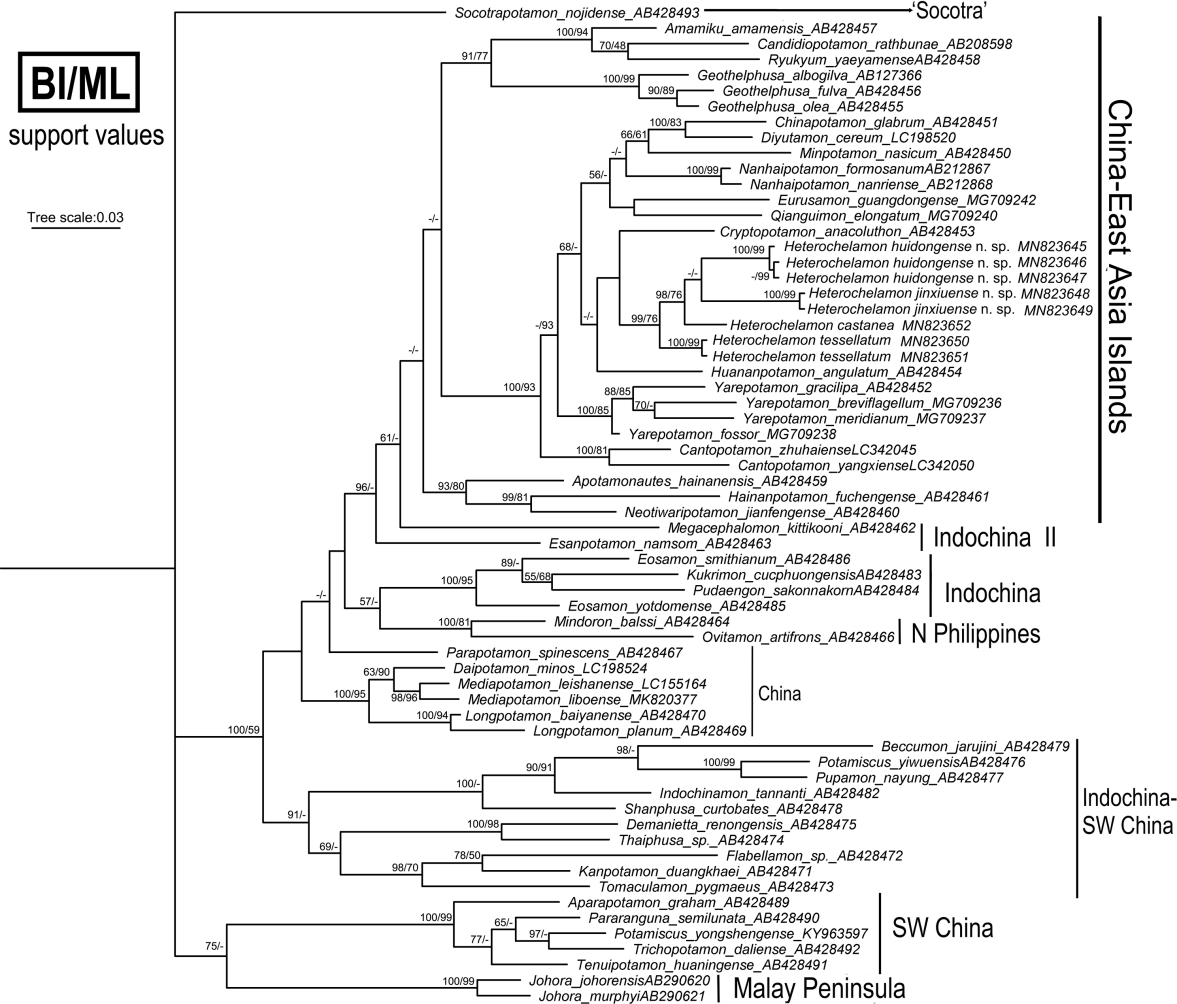
Phylogenetic tree. Reconstructed base on the 16S rRNA genes of *Heterochelamon* and some species of Asian potamids sampled for comparison. Branch lengths and topologies were obtained from BI analysis. Probability values at the nodes represent support values for BI and ML. Only values > 50% are shown. Photo credit: Song-Bo Wang.

We obtained the mitochondrial 16S rRNA gene molecular data from four species of *Heterochelamon* for this study. Other sequences used were downloaded from GenBank. To show where these two new species fit within the Asian freshwater crab phylogeny, 60 species of 47 genera in the family Potamidae Ortmann, 1896, were used (Table 2). The trees were constructed via the BI and ML methods and showed a high degree of consistency, and the topological structure was determined from BI with support values from both methods (Fig. 10). The phylogenetic results support the recognition of the two new *Heterochelamon* species, *H. tessellatum* and *H. castanea* cluster with the two new species to form an independent branch in the “China-East Asia Islands” clade (Shih, Yeo & Ng, 2009). It is possible that the two new species are in sister relationship. *H. castanea* is most closely related to the two new species in the two known congeneric species available for the molecular phylogenetic analysis.

**Table 2:** GenBank accession number of the species used for phylogenetic analysis. The 16S rRNA gene of 60 species belonging to 47 genera of the family Potamidae.

**Species**	**MuseumCatalogue No.**	**Locality**	**GenBank No.**
*Heterochelamon huidongense* n. sp.	NCU MCP 423604	Guangdong, China	MN823645
*Heterochelamon huidongense* n. sp.	NCU MCP 423605	Guangdong, China	MN823646
*Heterochelamon huidongense* n. sp.	NCU MCP 423606	Guangdong, China	MN823647
*Heterochelamon jinxiuense* n. sp.	NCU MCP 342004	Guangdong, China	MN823648
*Heterochelamon jinxiuense* n. sp.	NCU MCP 342005	Guangdong, China	MN823649
*Heterochelamon tessellatum*Naruse, Zhu & Zhou, 2013	NCU MCP 153601	Guangxi, China	MN823650
*Heterochelamon tessellatum*Naruse, Zhu & Zhou, 2013	NCU MCP 153602	Guangxi, China	MN823651
*Heterochelamon castanea*Naruse, Zhu & Zhou, 2013	NCU MCP 341901	Guangxi, China	MN823652
*Amamiku amamense* Minei, 1973	NCHUZOOL 13125	Amami, the Ryukyus	AB428457
*Aparapotamon grahami* Rathbun, 1929	ZRC YCM 0334(II)	Yunnan, China	AB428489
*Apotamonautes hainanensis* Parisi, 1916	ZRC	Hainan, China	AB428459
*Beccumon jarujini* Ng & Naiyanetr, 1993	ZRC 1991.1865 (paratype)	Chiangma, Thailand	AB428479
*Candidiopotamon rathbunae* De Man, 1914	NCHUZOOL	Nantou, Taiwan	AB208598
*Cantopotamon yangxiense* Chao, Ahyong & Shih, 2017	SYSBM 001564	Guangdong, China	LC342050
*Cantopotamon zhuhaiense* Chao, Ahyong & Shih, 2017	SYSBM 001439	Guangdong, China	LC342045
*Chinapotamon glabrum* Dai, Song, Li & Liang, 1980	CAS CB	Guangxi, China	AB428451
*Cryptopotamon anacoluthon* Kemp, 1918	NCHUZOOL 13122	Hong Kong	AB428453
*Daipotamon minos* Ng & Trontelj	ZRC 1996.1045	Guizhou, China	LC198524
*Demanietta renongensis* Rathbun, 1905	ZRC 1998.146	Ranong, Thailand	AB428475
*Diyutamon cereum* Huang, Shih & Ng	SYSBM	Guizhou, China	LC198520
*Eosamon smithianum* Kemp, 1923	ZRC	Chantaburi, Thailand	AB428486
*Eosamon yotdomense* Naiyanetr, 1984	ZRC 1991.1851	Ubon Ratchathani, Thailand	AB428485
*Esanpotamon namsom* Naiyanetr & Ng, 1997	ZRC 1997.776 (paratype)	Udon Thani, Thailand	AB428463
*Eurusanm guangdongense*Huang, 2018	SYSBM 001408	Guangxi, China	MG709242
*Flabellamon* sp.	ZRC	Mae Sot, Thailand	AB428472
*Geothelphusa albogilva* Shy, Ng & Yu, 1994	NCHUZOOL	Pingtung, Taiwan	AB127366
*Geothelphusa marginata fulva* Naruse, Shokita & Shy, 2004	NCHUZOOL 13124	Iriomote, the Ryukyus	AB428456
*Geothelphusa olea* Shy, Ng & Yu, 1994	NCHUZOOL 13123	Taichung, Taiwan	AB428455
*Hainanpotamon fuchengense* Dai, 1995	NCHUZOOL 13128	Hainan, China	AB428461
*Huananpotamon angulatum* Dai & Lin, 1979	ZRC	Fujian, China	AB428454
*Indochinamon tannanti* Rathbun, 1904	ZRC 1998.264	Yunnan, China	AB428482
*Johora johorensis* Roux, 1936	ZRC 1990.576	Gunung Pulai, Johor, Malaysia	AB290620
*Johora murphyi* Ng, 1986	ZRC 2001.2267	Kota Tinggi, Johor, Malaysia	AB290621
*Kanpotamon duangkhaei* Ng & Naiyanetr, 1993	ZRC	Kanchanaburi, Thailand	AB428471
*Kukrimon cucphuongense* Dang, 1975	ZRC NHH9729 160997	Ninh Binh, Vietnam	AB428483
*Longpotamon baiyanense* Ng & Dai, 1997	ZRC	Hunan, China	AB428470
*Longpotamon planum* Dai, 1992	ZRC 1998.1178	Anhui, China	AB428469
*Mediapotamon leishanense* Dai, 1995	SYSBM 001094	Guizhou, China	LC155164
*Mediapotamon liboense*Wang, Zhou & Zou, 2019	NCU MCP 343004	Guizhou, China	MK820377
*Megacephalomon kittikooni* Yeo & Naiyanetr, 1999	ZRC 1998.22 (holotype)	Xieng Khuang, Laos	AB428462
*Mindoron balssi* Bott, 1968	ZRC	Mindoro, the Philippines	AB428464
*Minpotamon nasicum* Dai & Chen, 1979	NCHUZOOL 13121	Fujian, China	AB428450
*Nanhaipotamon formosensis* Parisi, 1916	NCHUZOOL 13144	Tainan, Taiwan	AB212867
*Nanhaipotamon nanriense* Dai, 1997	CAS CB05103	Fujian, China	AB212868
*Neotiwaripotamon jianfengense* Dai & Naiyanetr, 1994	NCHUZOOL 13127	Hainan, China	AB428460
*Ovitamon artifrons* Bürger, 1894	ZRC	Luzon, the Philippines	AB428466
*Parapotamon spinescens* Calman, 1905	NCU MCP	Yunnan, China	AB428467
*Pararanguna semilunatum* Dai & Chen, 1985	ZRC	Yunnan, China	AB428490
*Potamiscus yiwuensis* Dai & Cai, 1998	ZRC	Yunnan, China	AB428476
*Potamiscus yongshengense* Dai & Chen, 1985	NNU150951	Yunnan, China	KY963597
*Pudaengon sakonnakorn* Ng & Naiyanetr, 1995	ZRC	Thailand	AB428484
*Pupamon nayung* Naiyanetr, 1993	ZRC 1995.558 (paratype)	Udon Thani, Thailand	AB428477
*Qianguimon elongatum*Huang, 2018	SYSBM 001424	Guangdong, China	MG709240
*Ryukyum yaeyamense* Minei, 1973	NCHUZOOL 13126	Iriomote, the Ryukyus	AB428458
*Shanphusa curtobates* Kemp, 1918	NRM 13920	Taunggyi, Shan State, Myanmar	AB428478
*Socotrapotamon nojidensis* Apel & Brandis, 2000	ZRC 2000.2232	Socotra, Yemen	AB428493
*Tenuipotamon huaningense* Dai & Bo, 1994	CAS CB05175	Yunnan, China	AB428491
*Thaiphusa* sp.	ZRC 1997.656	Thailand	AB428474
*Tomaculamon pygmaeus* Yeo & Ng, 1997	ZRC 1997.326-330 (paratype)	Phitsanulok, Thailand	AB428473
*Trichopotamon daliense* Dai & Chen, 1985	NCHUZOOL 13130	Yunnan, China	AB428492
*Yarepotamon breviflagellum* (Huang, 2018)	SYSBM 001442	Guangdong, China	MG709236
*Yarepotamon fossor* (Huang, 2018)	SYSBM 001417	Guangxi, China	MG709238
*Yarepotamon gracilipa* Dai, Song, Li & Liang, 1980	ZRC	Guangxi, China	AB428452
*Yarepotamon meridianum*Huang, 2018	SYSBM 001581	Guangdong, China	MG709237

**Notes.**

CAS CB Chinese Academy of Sciences, Beijing, China NCHUZOOL Zoological Collections of the Department of Life Science, National Chung Hsing University, Taichung, Taiwan NCU MCP, Department of Parasitology of the Medical College of Nanchang University, Jiangxi, China NNU College of Life Sciences, Nanjing Normal University, Nanjing, China NRM Swedish Museum of Natural History, Stockholm, Sweden SYSBM Sun Yat-sen Museum of Biology, Sun Yat-Sen University, Guangzhou, China ZRC Zoological Reference Collection of the Raffles Museum of Biodiversity Research, National University of Singapore, Singapore

## Discussion

The genetic analysis supports the recognition of four species of *Heterochelamon* which can also be differentiated morphologically. These four species form a monophyletic group with relatively high support values. Due to the lack of specimens of the other three species of *Heterochelamon*, we were unable to obtain their molecular data for analysis and therefore unable to determine the phylogenetic relationships of these species with the above four species.

Phylogenetically, it is possible that *Heterochelamon huidongense* n. sp. is more closely related to *Heterochelamon jinxiuense* n. sp. than any other congener (Fig. 10). However, the two can be immediately separated by the following characters: the epibranchial tooth of *H. huidongense* is very sharp (versus blunt in *H. jinxiuense*) (Figs. 1A and 6A), adult male chelipeds of *H. huidongense* unequal (versus strongly unequal in *H. jinxiuense*) (Figs. 1A and 6A), G1 terminal segment of *H. huidongense* bent in middle and not tapered distally (versus straight and tapered distally in *H. jinxiuense*) (Figs. 4A and 9A). The detailed differences among the two new species and other congeneric species are also presented in Table 1.

Türkay & Dai (1997) established *Heterochelamon* with *Potamon (Geothelphusa) pupureomanualis* (Wu, 1934) as a type species. However, the real identity of the specimens examined by Türkay & Dai (1997) is questionable, because their specimens are different from the original description by Wu (1934) in the following particulars, although we could not examine type material or topotypic material from Luocheng Country, Guangxi Zhuang Autonomous Region: the epibranchial tooth is very sharp in the type material (Wu, 1934) versus blunt in the specimens examined by Türkay & Dai (1997), and the G1 terminal segment has a bent tip in the type material (Wu, 1934) versus straight terminal segment in the specimens examined by Türkay & Dai (1997) (see also Table 1). Furthermore, the specimens examined by Türkay & Dai (1997) were said to be collected from Xiushui County, Guangxi. The locality record seems to be incorrect, because Xiushui County is in Jiangxi, not in Guangxi. Dai (1999) corrected the locality of the specimens studied by Türkay & Dai (1997) to Xiuren County, Guangxi, and we believe that this is correct locality. We concur with Naruse, Zhu & Zhou (2013) that the specimens referred to *H. pupureomanualis* by Türkay & Dai (1997) could actually represent an undescribed species. This matter can only to be addressed when the specimens can be re-examined.

With a few exceptions, most Chinese freshwater crab genera have restricted distributional ranges. The geographical distribution pattern of many freshwater crabs is caused by geographical isolation, which is due to the limited dispersal abilities of freshwater crabs and low fertility (Yeo et al., 2008; Ji et al., 2016; Jia et al., 2018). The distribution pattern of freshwater crabs in mainland China is closely related to the regional differentiation of China’s natural geographical environment. *Heterochelamon* has been reported to be mainly distributed in northern Guangxi (Türkay & Dai, 1997; Naruse, Zhu & Zhou, 2013). Noteworthy in this paper is that we describe a new species assigned to the genus from southern Guangdong, which is not in the known range of the genus, suggesting that it has a wider distributional pattern than previously thought. That being said, the distribution of *Heterochelamon* is still within the Huanan freshwater zoogeographical province, which mainly consists of Guangxi, Guangdong and Hainan (Huang, Ebach & Ahyong, 2020). Within this zoogeographical province, there are relatively few abiotic factors such has high mountains and rivers that may obstruct the dispersal of freshwater crabs. We therefore believe that there are other new species of *Heterochelamon* in this zoogeographical province.

## Conclusions

This study describes two new species of freshwater potamid crabs, referred to the genus *Heterochelamon*. We obtained sequence data of the mitochondrial 16S rRNA gene from the two new species and two other congeneric species for genetic analysis. The analysis supports the recognition of the two new species and the monophyly of the genus. The known distribution of this genus has been heretofore restricted to northern Guangxi, but our study now shows that the genus also occurs in southern Guangdong. (GenBank: MN823645 to MN823652).

##  Supplemental Information

10.7717/peerj.9565/supp-1 Supplemental Information 1 The 16S rRNA gene sequence upload in this study

10.7717/peerj.9565/supp-2 Supplemental Information 2 The original BI tree

10.7717/peerj.9565/supp-3 Supplemental Information 3 The original ML tree

## Additional Information and Declarations

### DNA Deposition

The following information was supplied regarding the deposition of DNA sequences: The sequences of the four species of the genus Heterochelamon are available at GenBank: MN823645 to MN823652.

### Data Availability

The following information was supplied regarding data availability: The raw measurements are available in the Supplementary Files. The raw data shows the news sequences uploaded in this study and the original phylogenetic trees used for phylogenetic analyses. The specimens were deposited in the Department of Parasitology of the Medical College of Nanchang University, Jiangxi, China (NCUMCP), National Tropical Disease Research Center, Shanghai, China (TDRC). The accession numbers for each specimen of Heterochelamon huidongense n. sp. are as follows: Holotype: NCU MCP 423601; Paratypes: NCU MCP 423602, NCU MCP 423603, TDRC 002005, TDRC 002006, TDRC 002007; Others: NCU MCP 423606, NCU MCP 423607, NCU MCP 423608, NCU MCP 423609, NCU MCP 423610, NCU MCP 423612, NCU MCP 423613. The accession numbers for each specimen of Heterochelamon jinxiuense n. sp. are as follows: Holotype: NCU MCP 342001; Paratypes: NCU MCP 342002, NCU MCP 342003, TDRC 002008, TDRC 002009; Other: NCU MCP 342006.

### New Species Registration

The following information was supplied regarding the registration of a newly described species: Publication LSID: urn:lsid:zoobank.org:pub:F680C21E-0BED-4724-BA62-1174336F433F Heterochelamon huidongense n. sp. LSID: urn:lsid:zoobank.org:act:0F67FA91-6734-4800-9C03-76668635A1E2. Heterochelamon jinxiuense n. sp. LSID: urn:lsid:zoobank.org:act:5D34B999-78C7-4522-9F74-E872A34B7462.
